# Necrotizing enterocolitis: risk factors and predictive modeling in a cohort of preterm infants. A case-control study

**DOI:** 10.3389/fped.2026.1880723

**Published:** 2026-07-16

**Authors:** T. Pérez-Oliver, A. Pinilla-Gonzalez, M. Gormaz, J. Kuligowski, E. Serna, L. Torrejón-Rodríguez, I. Lara-Cantón, A. Cayuela-Hernández, M. Vento, A. Parra-Llorca, M. Cernada

**Affiliations:** 1 Neonatal Research Group, Health Research Institute La Fe (IISLAFE), Valencia, Spain; 2 Division of Neonatology, La Fe University and Polytechnic Hospital (HULAFE), Valencia, Spain; 3 Spanish Network in Maternal, Neonatal, Child and Developmental Health Research (RICORS-SAMID) (RD24/0013/0014), Neonatal Research Group, Health Research Institute La Fe, Valencia, Spain; 4 Department of Physiology, Facultat de Medicina i Odontologia, Universitat de Valencia, Valencia, Spain; 5 MODULAhR Group, Universitat de Valencia, Valencia, Spain; 6 Centro de Investigación Biomédica en Red Fragilidad y Envejecimiento Saludable CIBERFES, INCLIVA Biomedical Research Institute, Valencia, Spain

**Keywords:** case-control study, necrotizing enterocolitis, predictive modeling, preterm infants, risk factors, very low birth weight

## Abstract

**Background:**

Necrotizing enterocolitis (NEC) is a leading cause of morbidity and mortality in preterm infants. Multiple risk factors have been reported, with inconsistent findings across studies. This study aimed to identify perinatal and postnatal risk factors associated with NEC in very preterm infants and to develop a predictive model for clinical use.

**Methods:**

We conducted a retrospective case-control study including infants <32 weeks’ gestation and <1,500 g birth weight admitted to a level III NICU between 2018 and 2022. NEC was defined as stage IIA or higher according to modified Bell's criteria and diagnosed within the first 65 days of life. Demographic, clinical, and laboratory data were analysed. Multivariable logistic regression was used to identify independent predictors of NEC and to generate a nomogram.

**Results:**

Of 354 eligible infants, 46 (13%) developed NEC. NEC was significantly associated with lower gestational age and birth weight, prolonged rupture of membranes, maternal and neonatal antibiotic exposure, use of umbilical arterial catheters, vasoactive drugs, feeding intolerance, anaemia, and platelet transfusion. In multivariable analysis, early intravenous antibiotic administration within the first 24 h of life and placental abruption remained independent risk factors for NEC (OR: 1.91, 95% CI: 1.1–3.3; and OR: 2.72, 95% CI: 1.27–6.14, respectively). The predictive model demonstrated moderate discriminatory ability (AUC = 0.73, 95% CI: 0.68–0.78).

**Conclusion:**

Early intravenous antibiotic administration and placental abruption were independently associated with NEC in very preterm infants. The proposed nomogram may support early risk stratification and closer clinical surveillance using readily available clinical variables. Further multicentre studies are required to validate this predictive tool and assess its clinical utility.

## Introduction

Each year, approximately 13.4 million babies are born prematurely worldwide (1). In high-income countries, complications secondary to preterm birth are the leading cause of neonatal death. Necrotizing enterocolitis (NEC) remains one of the neonatal complications with a higher incidence (7%–12%) and mortality rate, ranging between 14%–30% (2). Despite overall improvements in neonatal intensive care and survival, the incidence and mortality rates have remained unchanged over recent decades (3).

NEC is a severe inflammatory condition that leads to intestinal ischaemia, necrosis, and bowel perforation in advanced stages, affecting up to 8% of neonates admitted to intensive care units (3). The pathophysiology of NEC is multifactorial and remains poorly understood (4). Multiple risk factors have been reported, with inconsistent data across studies, likely due to confounding factors and/or differences in study design, populations, and demographic or environmental factors (4).

We hypothesised that modifiable perinatal and postnatal factors contribute to the risk of NEC. A retrospective case–control study was conducted to evaluate the impact of previously reported risk factors for NEC in preterm infants. The objective was to identify potentially modifiable perinatal and postnatal risk factors associated with NEC, intending to develop preventive strategies and optimize neonatal care practices and policies.

To address this objective, we included preterm infants born at <32 weeks' gestation (GW) and with a birth weight (BW) < 1,500 g born in a Regional Referral Centre (Hospital Universitario y Politécnico La Fe, Valencia) during a five-year period.

## Methods

### Research design

**Figure 1:**
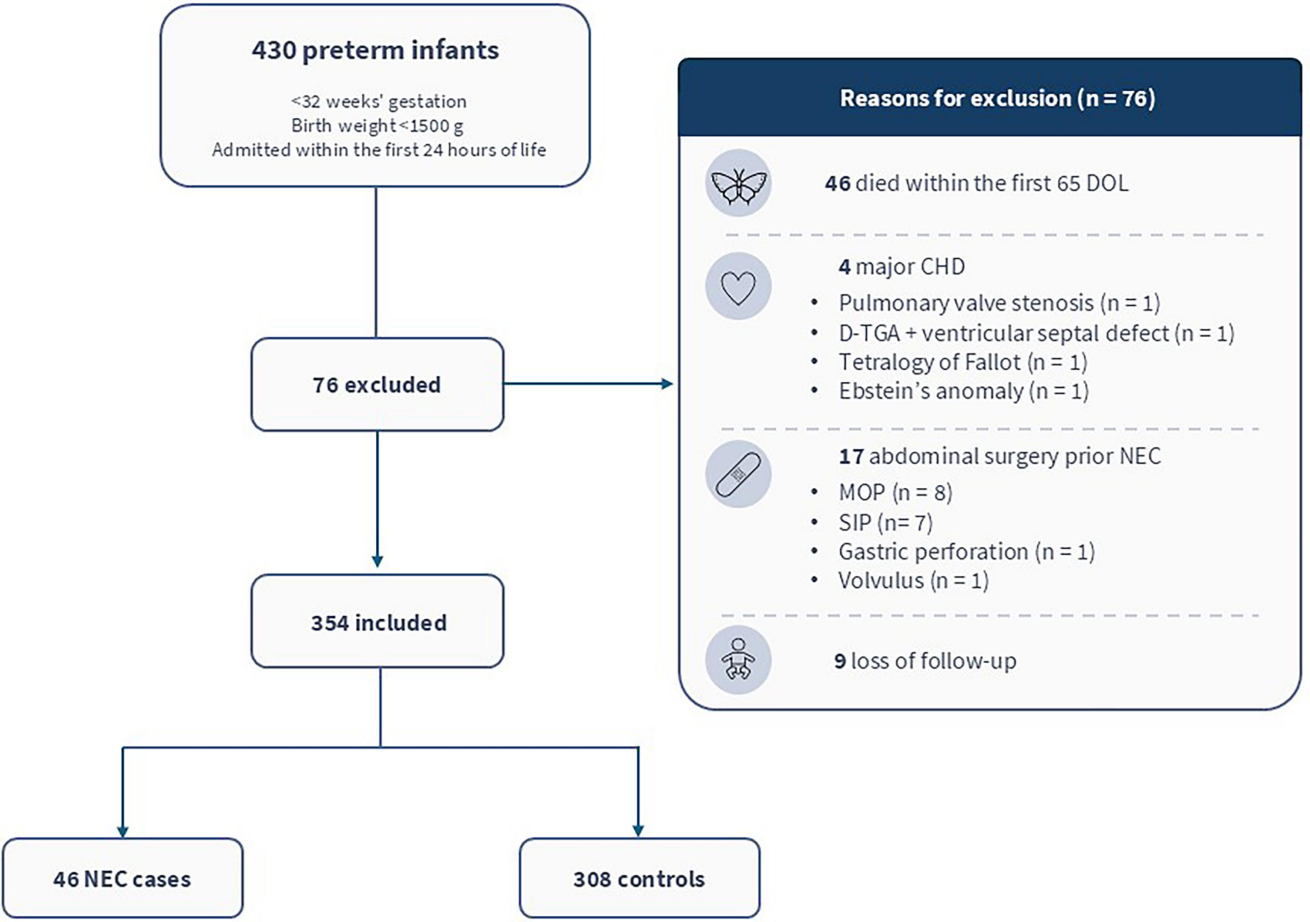
Flow diagram of patient enrolment in the study. 430 preterm infants born at <32 weeks of gestation and with a birth weight < 1,500 g between January 2018 and December 2022 were screened. The diagram shows the number of patients at each stage of selection, including exclusions and final cohort included in the analysis (*n* = 354). GW, weeks of gestation; BW, birth weight; DOL, days of life; CHD, congenital heart disease; D-TGA, transposition of great arteries; MOP, meconium obstruction of prematurity; SIP, spontaneous intestinal perforation.

This is a retrospective observational case-control study conducted at the Division of Neonatology of the La Fe University and Polytechnic Hospital (Valencia, Spain). We included all infants born at <32 GW and <1,500 g of BW who were admitted to our NICU between January 2018 and December 2022. The sample size was determined by including all the eligible admissions (Figure 1). NEC cases were infants who developed NEC of modified Bell stage IIA or higher (5) within the first 65 days of life (DOL) or before discharge. This time window was selected based on the known epidemiology of necrotizing enterocolitis in very preterm infants, in which onset is concentrated in the early postnatal period, with reported median ages of diagnosis ranging from approximately 2 to 3 weeks of life, while later presentations are uncommon but have been described in a minority of cases (6–8). Newborn infants who did not develop NEC served as controls.The study was approved by La Fe's Scientific and Ethics Committee for Biomedical Research (approval ID 2022-753-1), and all procedures were conducted in accordance with the Declaration of Helsinki. The requirement for informed consent was waived by the Ethics Committee due to the retrospective nature of the study and the use of anonymised routinely collected clinical data.

### Participants

Inclusion criteria: preterm babies born at <32 GW and with a BW < 1,500 g, admitted to our NICU, either born in our hospital or transferred from other centres.

Exclusion criteria: Infants who were transferred to our unit after 24 h of life; those who died within the first 65 days of life (DOL) (excluding deaths attributable to NEC); infants with major congenital heart disease (CHD); those who underwent abdominal surgery prior to NEC diagnosis, discharge or 65 DOL (whichever occurred first); and infants lost to follow-up due to transfer to other hospitals where outcome data were not available.

Inclusion and exclusion criteria are detailed in Supplementary Table 1 in the Supplementary file.

All eligible NEC cases and controls meeting inclusion criteria were included. Cases and controls were drawn from the same source population of preterm infants admitted to a single tertiary NICU during the study period, ensuring comparability and minimising selection bias. Controls consisted of infants who did not develop NEC during the predefined risk period (first 65 days of life or until discharge). No individual matching was performed; however, both groups were defined within the same at-risk cohort and time frame.

### Data collection procedures and variables' definition

Demographic, perinatal, clinical, and laboratory data were collected from patients' electronic medical records using a structured data collection form. Investigators had complete access to the hospital's records for all eligible infants. Data were checked for completeness and consistency. No linkage with other databases was performed, and all data came from a single institutional electronic record system. Patient confidentiality was maintained throughout the study.

The variables were classified as perinatal or postnatal data. Pathological prenatal situations were coded according to the obstetricians' diagnoses, the definition of the Spanish Obstetric Society (SEGO) and Spanish Neonatal Society, and postnatal pathologies were diagnosed according to standard definitions commonly used in neonatal practice. All variables were derived from routine clinical, laboratory, or radiological assessments, using identical measurement methods for cases and controls to ensure comparability. Post-diagnostic treatment variables (medical or surgical management of NEC) were not included, as the aim of the study was early pre-NEC risk prediction and only pre-diagnostic exposures were considered.

All clinical and laboratory time-dependent variables were restricted to pre-defined exposure windows to ensure comparability between groups, reflecting conditions prior to NEC onset in the affected group or within the first 65 days of life (or until discharge, whichever occurred first) in controls. This restriction applied to all relevant exposures, including laboratory measurements and transfusion events.

Laboratory variables were defined according to specific time windows. Umbilical arterial and venous pH values were obtained at birth as part of routine perinatal assessment. Minimum base excess was calculated from blood gas analyses performed within the first 12 h of life. Hemoglobin values were included only when obtained within the same pre-defined temporal framework. Red blood cell and platelet transfusions were recorded as binary variables (yes/no) and considered present if administered within the same exposure window.

As variables were defined directly from clinical criteria rather than administrative codes, a complete code list was not applicable. To minimise potential sources of bias, strict inclusion criteria and uniform diagnostic definitions were applied, and a standardised methodology ensured consistency in data extraction. Definitions of all variables are provided in Supplementary Table 2 in the Supplementary file.

### Data analysis

Quantitative variables were analysed as continuous measures and are presented as medians with interquartile ranges. No categorisation of continuous variables was applied. Univariate analysis was performed using Fisher's exact test for categorical variables and Mann–Whitney U test for the continuous ones. The Mann–Whitney U test was chosen for its robustness to non-normal distributions, and its applicability to approximately normal data. Variables showing statistical significance in univariate analysis, together with variables considered clinically relevant, were included in the multivariable logistic regression model to identify independent predictors of NEC while controlling for potential confounders. Logistic regression was used as the outcome was defined as a binary event (NEC vs. no NEC), and the aim of the study was risk prediction rather than time-to-event analysis. Model performance was evaluated using the area under the receiver operating characteristic (ROC) curve (AUC), and a nomogram was constructed to estimate individual risk of NEC. The nomogram was internally validated using leave-one-out cross-validation to reduce potential overfitting. Given the limited number of NEC cases (*n* = 46), subgroup and sensitivity analyses were not performed, as these analyses would likely have resulted in insufficient statistical power and unstable estimates. Statistical significance was set at a *p*-value < 0.05.

## Results

### Study population and NEC prevalence

Four hundred and thirty infants met the inclusion criteria. Seventy-six were excluded based on predefined exclusion criteria (Figure 1). A total of 354 infants were included in the statistical analysis, of whom 46 developed NEC, resulting in an overall incidence of 13% during the 5-year study period.

### Perinatal and postnatal characteristics

Infants who developed NEC were born at a lower GA [median 27 weeks [IQR: 25–29] vs. 29 weeks [27–30]; *p* < 0,001] and had lower BW [870 g [726–1,191] vs. 1,090 g [900–1,300]; *p* < 0,001] compared with controls. Maternal antibiotic use during pregnancy was more frequent in NEC cases (69,6% vs. 47,1%; *p* = 0.007) (Table 1). Most infants received antenatal steroids and magnesium sulphate, were delivered by C-section, and required basic resuscitation in the delivery room. Umbilical artery catheters (UACs) were used more frequently in infants with NEC (63% vs. 36%; *p* = 0.002), whereas the use of UVCs was similar (97.8% vs. 93.2%, *p* = 0.2) (Table 2). Moreover, NEC patients received antibiotics within the first 24 h of life more often (37% vs. 19.8%, *p* = 0,02), and their cumulative antibiotic exposure was higher [5 [IQR: 0–9] vs. 0 days [IQR: 0–7], *p* = 0,005]. They also showed poorer feeding tolerance on days 7 and 14 and achieved full enteral feeds approximately one day later than controls [11 [IQR: 9–20] vs. 10 days [IQR: 8–12], *p* = 0,003]. Vasoactive drugs were used more often in the NEC group (26.1% vs. 10.7%, *p* = 0.03).

**Table 1:** Perinatal characteristics in the NEC and control groups.

Variable	Control (*n* = 308)	NEC (*n* = 46)	*p*-value
Gestational factors			
GA weeks, median (IQR)	29 (27–30)	27 (25–29)	**<0** **.** **001**
BW g, median (IQR)	1,090 (900–1,300)	870 (726–1,191)	**<0** **.** **001**
BW z-score, median (IQR)	−0.35 (−0.93–0.21)	−0.2 (−0.98–0.26)	0.804
Birth length in cm, median (IQR)	37 (34–39)	33 (31–36)	**<0** **.** **001**
Birth head circumference in cm, median (IQR)	26 (24.5–27)	24 (22.6–26)	**<0** **.** **001**
Male sex, *n* (%)	181 (58.8)	29 (63)	0.697
Singleton pregnancy, *n* (%)	193 (62.7)	28 (60.9)	0.368
TTTS, *n* (%)	22 (7.2)	6 (13)	0.563
No IUGR, *n* (%)	217 (70.5)	33 (71.7)	0.594
IUGR, *n* (%)	91 (29.5)	13 (28.3)	
Pathological Doppler, *n* (%)	94 (30.5)	10 (21.7)	0.296
Maternal and pregnancy related factors			
Mild PET, *n* (%)	57 (18.5)	5 (10.9)	0.444
HELLP syndrome, *n* (%)	13 (4.2)	2 (4.3)	
Placental abruption, *n* (%)	23 (7.5)	8 (17.4)	0.052
Suspected chorioamnionitis, *n* (%)	46 (15)	10 (21.7)	0.341
Maternal IVAB, *n* (%)	145 (47.1)	32 (69.6)	**0** **.** **007**
ROM in hours, median (IQR)	0 (0–4)	0 (0–120)	**0** **.** **028**
Antenatal corticosteroid treatment, *n* (%)	247 (80.2)	40 (87)	0.328
Incomplete antenatal corticosteroid course, *n* (%)	49 (15.9)	6 (13)	
Magnesium sulphate, *n* (%)	274 (89)	40 (87)	0.880
Perinatal factors			
C-section, *n* (%)	239 (77.6)	37 (80.4)	0.808
No resuscitation at delivery, *n* (%)	21 (6.8)	1 (2.2)	0.446
Basic resuscitation, *n* (%)	260 (84.4)	40 (87)	
Advanced resuscitation, *n* (%)	27 (8.8)	5 (10.9)	
Apgar @ 1 min, median (IQR)	8 (6–9)	7 (5–8)	0.091
Apgar @ 5 min, median (IQR)	9 (8–10)	9 (8–9)	0.099
Venous cord gas, median (IQR)	7.34 (7.28–7.38)	7.35 (7.3–7.38)	0.883
Arterial cord gas, median (IQR)	7.3 (7.23–7.33)	7.29 (7.21–7.35)	0.862

GA, gestational age; BW, birth weight; TTTS, twin to twin transfusion syndrome; IUGR, intrauterine growth restriction; PET, preeclampsia; HELLP, haemolysis, elevated liver enzymes, and low platelet count; IVAB, intravenous antibiotics; ROM, rupture of membranes.Bold values indicate statistically significant results (*p* < 0.05).

**Table 2:** Postnatal characteristics in the NEC and control groups.

Variable	Control (*n* = 308)	NEC (*n* = 46)	*p*-value
Temperature on admission °C, median (IQR)	36.4 (36–36.7)	36.5 (36.1–36.7)	0.574
Min. BE mEq/L, median (IQR)	−3.6 (−5.62– −2.08)	−3.6 (−5.2– −2.1)	0.905
Well-positioned UVC, *n* (%)	185 (60.1)	24 (52.2)	0.164
Suboptimal UVC, *n* (%)	102 (33.1)	21 (45.8)	
Days of UVC, median (IQR)	5 (4–6)	5 (4–6)	0.556
UAC, *n* (%)	112 (36.4)	29 (63)	**0** **.** **002**
Days of UAC, median (IQR)	0 (0–2)	2 (0–4)	**<0** **.** **001**
Did not require surfactant, *n* (%)	172 (55.9)	21 (45.7)	0.303
Single dose, *n* (%)	110 (35.7)	20 (43.5)	
Two or more doses, *n* (%)	26 (8.4)	5 (10.8)	
IVAB started on 1st DOL, *n* (%). Of which:	61 (19.8)	17 (37)	**0** **.** **015**
Ampicillin + Amikacin, *n* (%)	25 (41)	7 (41.2)	0.901
Ampicillin + cefotaxime, *n* (%)	34 (55.7)	10 (58.8)	
Other, *n* (%)	2 (3.2)	0 (0)	
IVAB therapy days, median (IQR)	0 (0–7)	5 (0–9)	**0** **.** **005**
Weight loss %, median (IQR)	10 (7–13)	11 (7–13)	0.923
MOP, *n* (%)	35 (11.4)	10 (21.7)	0.083
Enteral feeds on 7 DOL in mL/kg/day, median (IQR)	110 (60–130)	65 (10–120)	**<0** **.** **001**
Enteral feeds on 14 DOL in mL/kg/day, median (IQR)	180 (160–180)	125 (30–180)	**<0** **.** **001**
DOL when fully enteral fed, median (IQR). Of which:	10 (8–12)	11 (9–20)	**0** **.** **003**
MEBM	165 (55.2)	27 (60)	0.543
MEBM—DEBM	21 (7)	5 (11.1)	
DEBM	110 (36.8)	13 (28.9)	
Formula	3 (1)	0 (0)	
Significant PDA, *n* (%)	71 (23.1)	17 (37)	0.064
PDA treated with ibuprofen	44 (62)	10 (58.8)	1
Vasoactive drugs, *n* (%)	33 (10.7)	12 (26.1)	**0** **.** **007**
Corticosteroids, *n* (%)	28 (9.1)	12 (26.1)	**0** **.** **002**
Min. Hb in g/dL, median (IQR)	9.20 (8.40–10.00)	10.00 (8.80–12.73)	**0** **.** **001**
DOL at min. Hb, median (IQR)	34 (21–48)	17 (8–30)	**<0** **.** **001**
PRBC transfusion, *n* (%)	127 (41.2)	24 (52.2)	0.215
Platelet transfusion, *n* (%)	20 (6.5)	8 (17.4)	**0** **.** **024**

BE, base excess; UVC, umbilical venous catheter; UAC, umbilical arterial catheter; IVAB, intravenous antibiotics; DOL, days of life; MOP, meconium obstruction of prematurity; MEBM, mother's express breast milk; DEBM, donor's express breastmilk; PDA, persistent ductus arteriosus; Hb, haemoglobin; PRBC, packed red blood cells.Bold values indicate statistically significant results (*p* < 0.05).

**Figure 2:**
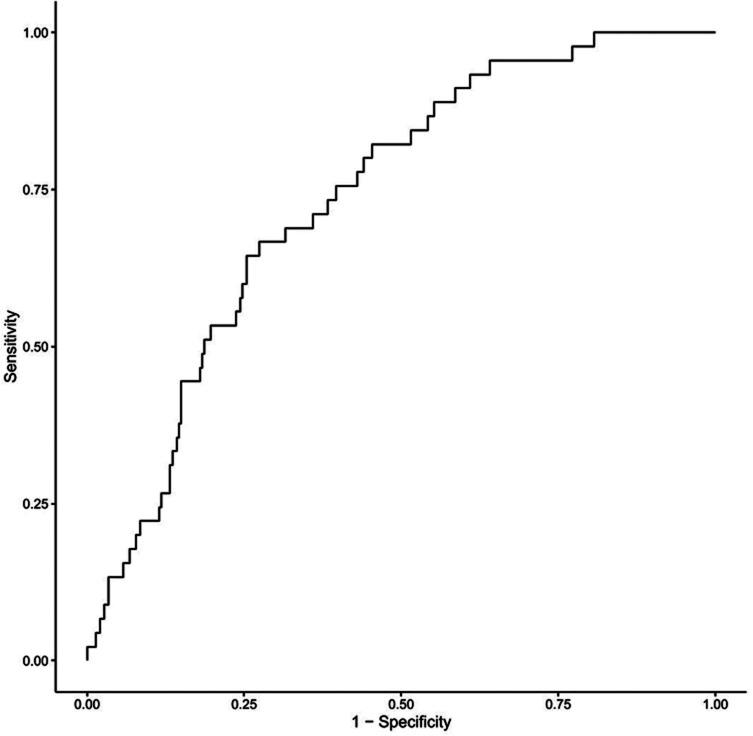
ROC curve of the multivariable logistic regression model for the prediction of NEC. The area under the curve (AUC) was 0.73 (95% CI: 0.68–0.78, calculated using the DeLong method), indicating moderate discriminatory ability.

**Figure 3:**
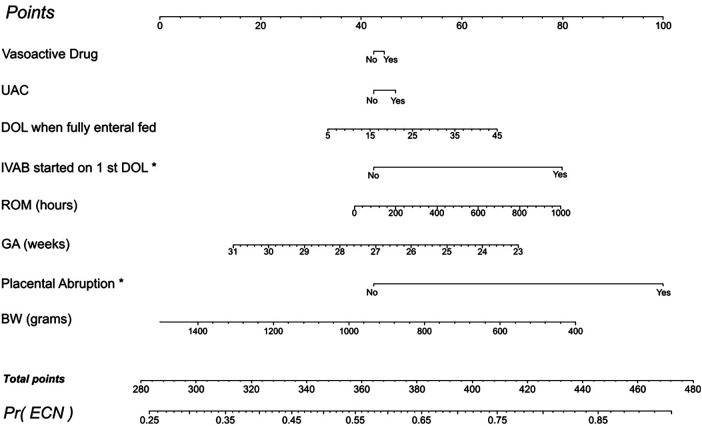
Nomogram model for predicting the occurrence of NEC in preterm babies < 32 weeks and <1,500 g during the first 65 days of life. Points are assigned based on the regression coefficients of each predictor, which are then summed to estimate the probability of NEC. UAC, umbilical arterial catheter; DOL, days of life; IVAB, intravenous antibiotics; ROM, rupture of membranes; GA, gestational age; BW, birth weight.

In the multivariable logistic regression model (Table 3), antibiotic treatment within the first day of life (OR: 1.91, *p* = 0.02) and placental abruption (OR: 2.72, *p* = 0.011) were independently associated with NEC. The model showed moderate discriminative performance, with an AUC of 0.73 (95% CI: 0.68–0.78, calculated using the DeLong method) (Figure 2). A nomogram was constructed from the model to estimate individual NEC risk using early clinical variables (Figure 3).

**Table 3:** Logistic regression.

Variable	Coefficient	C.I. 95%	Odds-ratio	*p*-value
Intercept	4.25	[−0.5, 8]	63.6	0.08
GA	−0.12	[−0.2, 0.05]	0.88	0.16
BW	−0.0013	[−0.002, 0.000004]	0.99	0.051
UAC	0.07	[–0.57, 0.7]	1.07	0.81
Vasoactive drug	0.036	[−0.79, 0.8]	1.03	0.93
Hours of ROM	0.0007	[−0.0003, 0.0019]	1.0007	0.22
IVAB 1st DOL	0.65	[0.1, 1.2]	1.91	**0** **.** **02**
Placental abruption	1.001	0.24, 1.8]	2.72	**0** **.** **011**
DOL when fully enteral fed	0.01	[–0.01, 0.005]	1.01	0.35

GA, gestational age; BW, birth weight; UAC, umbilical arterial catheter; ROM, rupture of membranes; IVAB, intravenous antibiotics; DOL: days of life.Bold values indicate statistically significant results (*p* < 0.05).

## Discussion

This study aimed to identify risk factors associated with stage IIA or higher NEC in preterm infants born at <32 GW and <1,500 g. NEC incidence was 13%, consistent with previously reported rates in very low birth weight infants (VLBW) (9–11). In univariate analyses, several factors were significantly associated with NEC, including lower GA and birth anthropometric measures; prolonged ROM; maternal and early neonatal antibiotic exposure; poorer early feeding tolerance; UAC use; use of vasoactive drugs; lower minimum Hb levels; platelet transfusion; and NAC administration. However, in the multivariate model, only placental abruption and antibiotic exposure within the first 24 h of life remained independently associated with NEC.

Consistent with prior studies, infants who developed NEC had lower gestational age and birth weight compared to those who did not develop NEC (4, 12, 13). Although previous research has shown that small for gestation age (SGA) and IUGR infants have approximately twice the risk of NEC (13–16), our cohort showed no association with BW z-score or IUGR, likely due to the small number of SGA and IUGR infants, which limited the statistical power to detect such associations.

Among perinatal factors, placental abruption emerged as the most significant risk factor. This finding is consistent with previous studies suggesting that compromised foetal perfusion may predispose to intestinal ischemia and NEC (12, 13). In contrast, maternal PET was not significantly associated with NEC, echoing mixed results reported in prior studies (13, 16).

We did not observe differences in Apgar scores or cord pH, suggesting that global perinatal depression may not play a major role in NEC in our population. This contrasts with previous observational studies reporting associations between indicators of poor perinatal transition and NEC (13, 17). Apgar scores < 7 at five minutes of life were associated with increased incidence of NEC in infants with GA > 32 weeks, but this association was not observed in more preterm infants (18). In our cohort, only a small proportion required advanced resuscitation (8.8% of controls; 10.9% of NEC cases), the mean 5 minute Apgar score was 9 in both groups, and all infants had an umbilical cord pH > 7.2.

Antenatal corticosteroids are known to reduce NEC incidence by up to 50%, a benefit confirmed in Cochrane reviews (9, 19–21). In our cohort, nearly all mothers received corticosteroids, limiting our ability to assess their specific effect on NEC risk due to the minimal number of unexposed infants.

Perinatal antibiotic therapies may disrupt the vertical transmission of beneficial microbiota and predispose infants to dysbiosis (16, 22). In our cohort, maternal antibiotic exposure and longer ROM were associated with NEC, which aligns with previous studies (23). However, the role of subclinical chorioamnionitis remains unclear. Despite known differences in maternal–infant microbiota transfer between vaginal and caesarean births (16), the association between caesarean delivery and NEC remains controversial (13, 18), and no differences were observed in our population, where nearly 80% were delivered by caesarean section. Antibiotic use is another major disruptor of the gut microbiome (16, 24) and both, IVAB within the first 24 h of life and longer antibiotic duration, were risk factors for NEC in our cohort. This is consistent with evidence showing increased NEC risk with antibiotic courses longer than 3 days (13, 20, 25), with one study reporting a 20% increase in the risk of NEC for each additional day (26, 27), and others linking prolonged early antibiotic exposure in VLBW infants to NEC, late-onset sepsis, and death (7, 26). Remarkably, early-onset sepsis was confirmed by blood culture in only 4 of the 17 patients treated upon admission.

Infants diagnosed with NEC showed poorer feeding tolerance at days 7 and 14 of life and reached full enteral nutrition later than controls, which is consistent with data published in previous studies (6, 28). Although reduced peristalsis could theoretically contribute to bacterial overgrowth and mucosal injury, this was not supported by our data, as NEC patients did not have a higher incidence of MOP. Similar studies have likewise found no association between NEC and other markers of gut dysmotility, such as time to first meconium passage or duration of meconium evacuation (28).

Umbilical lines are commonly used in VLBW infants, and some retrospective studies have suggested that they may disrupt intestinal blood flow and increase NEC risk (23). Moreover, mispositioned UVCs may potentially contribute to portal congestion and intestinal injury (10). In our cohort, UVC use—even when suboptimally positioned—was not associated with NEC, whereas the use of UAC was more frequent among NEC cases. Although the effects of UACs on intestinal perfusion remain debated, these catheters may promote microthrombi, splanchnic vasospasm, and reduced aortic lumen diameter, and higher or prolonged use (>5 days) has been linked to increased NEC risk (23).

PDA has traditionally been linked to NEC through impaired intestinal perfusion (6, 20), but recent evidence does not support PDA as a contributing factor (29). Studies report no differences in NEC incidence across infants without PDA, those with medically closed PDA, and those with persistent significant PDA (30), and the PDA-TOLERATE trial likewise found no difference between early treatment and conservative management (31). Our data show a non-significant trend toward PDA increasing the likelihood of NEC, and ibuprofen**—**the standard PDA therapy—was not associated with NEC, consistent with Cochrane findings (32, 33).

The use of vasoactive drugs was more common among infants with NEC. These agents may compromise splanchnic blood flow by prioritizing blood supply to vital organs (23). However, some theories suggest that hypotension itself, rather than the use of vasoactive drugs, is the actual risk factor for NEC (34).

Although anaemia has been proposed as a risk factor for NEC (13, 15), in our study Hb levels were higher in infants with NEC than in the control group. This finding is likely related to the timing of Hb measurement. Minimum Hb values occurred at a mean age of 17 days in the NEC group and 34 days in the control group. Because the physiological nadir occurs between 4 and 12 weeks of life in preterm infants (35), Hb in the control group was measured during this nadir, whereas in the NEC group it was measured earlier. It remains uncertain whether PRBC transfusion increases NEC risk in preterm infants, as existing evidence is graded “low” and “very low” (36). A recent meta-analysis show no increased risk of NEC (36), some studies suggest a protective effect (37), and others report an increased risk (11, 20, 25). Several studies have indicated that severe anaemia—rather than transfusion itself- is associated with an elevated risk of NEC, suggesting that preventing anaemia might be more beneficial than limiting PRBC transfusions (37, 38). In our population, PRBC transfusion was not identified a risk factor for NEC. However, we found this association with platelet transfusion. Although few studies have examined the relationship between platelet transfusion and NEC, a metanalysis reported evidence of such an association (39).

Breastfeeding has been consistently shown to decrease the risk of NEC (9, 13, 20, 25). Breast milk has lower osmolarity compared with formula and is also rich in secretory IgA, lactoferrin and other antimicrobial active substances (11, 20, 25). In our unit, when maternal breast milk is unavailable, donor breast milk is provided to VLBW infants. As a result, only 3 patients during the study period were fed with formula, which limits our ability to analyse the effect of human milk vs. formula on NEC incidence.

Our logistic regression model showed moderate predictive performance (AUC = 0.73), suggesting potential clinical utility. The nomogram provides an accessible visual tool to estimate individual NEC risk in infants <32 weeks and <1,500 g during the first 65 days of life, using early clinical variables. Its sensitivity and specificity (0.66 and 0.72, respectively) indicate a balanced discriminatory capacity and suggest that the model may help identify infants at increased risk while maintaining an acceptable rate of false positives. Although the model is not intended to function as a definitive diagnostic instrument, it may support early risk stratification and guide closer clinical surveillance in infants with higher predicted probabilities. The use of objective variables that are routinely collected in neonatal care also supports the feasibility of implementing the nomogram at the bedside.

Several nomograms have previously been proposed for NEC risk assessment. Some have been developed in specific clinical settings, such as early- or late-onset sepsis, limiting their applicability to selected subgroups of preterm infants (40, 41). Other models have focused on predicting outcomes after NEC diagnosis, including mortality, the need for surgical intervention, or postoperative complications such as intestinal failure (42–44). In contrast, our model is intended for early-life risk prediction, before the onset of clinical disease. This earlier application may allow closer monitoring and more timely clinical awareness in high-risk infants.

Notably, a multicenter study developed a nomogram for the early prediction of NEC in preterm infants using perinatal and clinical risk factors (45). While some predictors overlap with those identified in our cohort, others differ, reflecting the heterogeneity of NEC pathogenesis and the absence of a universally accepted risk profile. Collectively, these findings highlight the complexity of NEC risk stratification and support the need for further validation of predictive models across diverse neonatal settings. This study has several limitations. It is an observational, retrospective, single-centre study conducted in a high-income country, which may limit the generalizability of the findings to other centres and to low- and middle-income countries. Therefore, external validation of the nomogram is required. In addition, the limited sample size may have lacked sufficient power to detect associations for less frequent variables. Finally, although missing data were not formally recorded, unrecognised gaps in retrospective records cannot be excluded.

Despite these limitations, the study also presents several strengths. The relatively short study period reduced variability associated with changes in neonatal care practices, and all clinical data were retrieved by a single investigator, ensuring uniform interpretation of clinical parameters and reducing the likelihood of differential information bias.

Overall, these findings should be interpreted cautiously. Despite the retrospective, single-centre design and potential misclassification or unmeasured confounding that may have weakened some associations, the results align with existing evidence and add meaningful insight into early NEC risk. In this cohort, early intravenous antibiotic administration and placental abruption were independently associated with necrotizing enterocolitis, with placental abruption emerging as a potentially underrecognized prenatal contributor. These findings highlight the need to further explore early antibiotic exposure and placental pathology in larger multicentre studies and to externally validate the proposed predictive model.

## Data Availability

The raw data supporting the conclusions of this article will be made available by the authors, without undue reservation.
