# Supplementary material for: Necrotizing enterocolitis: risk factors and predictive modeling in a cohort of preterm infants. A case-control study
Source: Front Pediatr. 2026 Jul 16;14:1880723. doi: 10.3389/fped.2026.1880723 (PMC13422380; doi:10.3389/fped.2026.1880723)
Supplement: Supplementary file 1 [file Supplementaryfile1.docx]

**SUPPLEMENTARY TABLE 1.** INCLUSION AND EXCLUSION CRITERIA FOR THE STUDY.

| **Inclusion criteria** | **Exclusion criteria** |
| --- | --- |
| Born at <32 weeks’ gestational age | Transferred to our NICU after 24 hours of life |
| Birth weight <1500 g | Death within the first 65 days of life (excluding deaths attributable to NEC) |
| Admitted to our NICU | Major congenital heart disease |
| Inborn or transferred from other centres | Abdominal surgery prior to NEC diagnosis, discharge, or 65 days of life (whichever occurred first) |
|  | Lost of follow-up |

**SUPPLEMENTARY TABLE 2**. VARIABLE DEFINITIONS.

| **Variable** | **Definition** |
| --- | --- |
| **Gestational age** | Completed weeks of gestation at birth |
| **Birth weight** | First weight, in grams, measured after birth |
| **Birth length** | First length, in centimetres, measured after birth |
| **Birth head circumference** | First head circumference, in centimetres, measured after birth |
| **Singleton pregnancy** | Pregnancy with a single fetus, with no evidence of multiple gestation at any time during pregnancy |
| **Twin-to-twin transfusion syndrome** | Complication of monochorionic diamniotic twin pregnancies diagnosed by a polyhydramnios–oligohydramnios sequence on ultrasound |
| **Intrauterine growth restriction** | Prenatal diagnosis of impaired fetal growth based on estimated fetal weight below the 10th percentile for gestational age |
| **Pathological Doppler** | Abnormal fetal Doppler ultrasound findings involving one or more fetal vessels |
| **Preeclampsia** | Hypertensive disorder of pregnancy characterized by new-onset hypertension after 20 weeks of gestation |
| **HELLP syndrome** | Severe form of preeclampsia characterized by hemolysis, elevated liver enzymes, and low platelet count |
| **Placental abruption** | Premature separation of the placenta from the uterine wall before delivery |
| **Suspected chorioamnionitis** | Clinical diagnosis of chorioamnionitis based on maternal and/or laboratory findings, without microbiological confirmation |
| **Maternal intravenous antibiotics** | Administration of systemic intravenous antibiotics to the mother during pregnancy or delivery |
| **Rupture of membranes** | Time interval, in hours, from rupture of membranes to delivery |
| **Antenatal steroid treatment** | Administration of a complete antenatal corticosteroids course* for foetal lung maturation |
| **Incomplete steroid course** | Administration of less than a full recommended course of antenatal corticosteroids |
| **Magnesium sulphate** | Antenatal administration of magnesium sulphate for foetal neuroprotection |
| **Delivery room resuscitation** | Resuscitation measures required immediately after birth |
| **Basic resuscitation** | Initial resuscitation measures including non-invasive airway support |
| **Advanced resuscitation** | Resuscitation requiring endotracheal intubation, chest compressions, and/or administration of medications |
| **Apgar score** | Standard clinical assessment of neonatal condition at 1 and 5 minutes after birth |

**SUPPLEMENTARY TABLE 2 (CONTINUED)**. VARIABLE DEFINITIONS.

| **Variable** | **Definition** |
| --- | --- |
| **Venous cord gas** | pH measured from umbilical venous cord blood immediately after birth. |
| **Arterial cord gas** | pH measured from umbilical arterial cord blood immediately after birth. |
| **Temperature on admission** | Axillary temperature measured at NICU admission |
| **Minimum base excess** | Lowest base excess value recorded during the first 12 hours of life |
| **Well-positioned umbilical venous catheter** | Umbilical venous catheter with the tip in a central position at the inferior vena cava–right atrial junction confirmed by imaging |
| **Suboptimal umbilical venous catheter** | Umbilical venous catheter with the tip not located at the inferior vena cava–right atrial junction |
| **Days of umbilical venous catheter** | Number of days the umbilical venous catheter remained in situ |
| **Umbilical arterial catheter** | Umbilical arterial catheter with the tip located in a high position between thoracic vertebral levels T6 and T9^†^ |
| **Days of umbilical arterial catheter** | Number of days the arterial venous catheter remained in situ |
| **No Surfactant Required** | No administration of exogenous surfactant |
| **Single dose of surfactant** | Administration of one dose of exogenous surfactant^‡^ |
| **Two or more doses of surfactant** | Administration of two or three doses^‡^ of exogenous surfactant |
| **Intravenous antibiotics started on day one of life** | Initiation of intravenous antibiotic therapy on day 1 of life |
| **Intravenous antibiotics therapy days** | Cumulative days of intravenous antibiotic therapy, including multiple courses |
| **Weight loss** | Maximum percentage of weight loss from birth weight |
| **Meconium obstruction of prematurity** | Clinical diagnosis characterized by abdominal distension with dilated bowel loops, with or without vomiting or bilious gastric residuals, and signs of ileus preventing advancement of enteral feeding; abdominal radiographs show dilated intestinal loops |
| **Enteral feeds on day 7 and day 14 of life** | Daily enteral intake expressed in mL/kg on days 7 and 14 of life |
| **Day of life at fully enteral feeding** | Day of life when full enteral feeding^§^ was achieved |
| **Milk type at fully enteral feeding** | Type of milk^¶^ when full enteral feeding was achieved |

**SUPPLEMENTARY TABLE 2 (CONTINUED)**. VARIABLE DEFINITIONS.

| **Variable** | **Definition** |
| --- | --- |
| **Significant patent ductus arteriosus** | A ductus arteriosus confirmed by echocardiography and associated with clinical and echocardiographic signs of hemodynamic significance, requiring medical treatment |
| **Ibuprofen as patent ductus arteriosus treatment** | Pharmacological treatment of PDA with ibuprofen according to standard practice**^‖^** |
| **Vasoactive drugs** | Administration of vasoactive medications for cardiovascular support prior to NEC, 65 days of life or discharge (whichever occurred first) |
| **Corticosteroid treatment** | Systemic corticosteroid therapy administered prior to NEC, 65 days of life or discharge (whichever occurred first) |
| **Minimum hemoglobin** | Lowest hemoglobin value recorded prior to NEC, 65 days of life or discharge (whichever occurred first) |
| **Day of life at minimum hemoglobin** | Day of life when the lowest hemoglobin value was recorded |
| **Packed red blood cell transfusion** | Administration of packed red blood cell transfusion prior to NEC, 65 days of life or discharge (whichever occurred first) |
| **Platelet transfusion** | Administration of platelet transfusion prior to NEC, 65 days of life or discharge (whichever occurred first) |

*Complete course: intramuscular betamethasone 12 mg every 24 hours for 2 doses

^†^Umbilical arterial catheters were intended to be placed in a high position (T6–T9). Low-positioned catheters were routinely removed and therefore not maintained in situ.

^‡^First dose at 200 mg/kg. Second and third dose at 100 mg/kg

^§^Full enteral feeding was defined as enteral intake >150 mL/kg/day.

^¶^Milk type at full enteral feeding: Mother expressed breast milk (>80% mother’s own milk), mother expressed breast milk – donor expressed breast milk (50/50 mother’s own and donor milk), donor expressed breast milk (>80% donor milk), or formula (>80% formula).

**^‖^**Ibuprofen was administered according to institutional standard practice (10 mg/kg on day 1, followed by 5 mg/kg every 24 hours on days 2 and 3). A second course was given if the ductus remained patent and met treatment criteria
